# Clinical performance of the Femoral Neck System within 1 year in 125 patients with acute femoral neck fractures, a prospective observational case series

**DOI:** 10.1007/s00402-022-04686-w

**Published:** 2022-12-02

**Authors:** Karl Stoffel, Christian Michelitsch, Rohit Arora, Reto Babst, Christian Candrian, Alexander Eickhoff, Florian Gebhard, Andreas Platz, Florian Andreas Schmid, Wolfram Weschenfelder, Christoph Sommer

**Affiliations:** 1 Department of Orthopaedics and Traumatology, University Hospital Basel, Spitalstrasse 21, 4031 Basel, Switzerland; 2 Department of Surgery, Kantonsspital Graubuenden, Chur, Switzerland; 3 Department for Orthopaedics Trauma Surgery, Medical University Innsbruck, Innsbruck, Austria; 4 Department of Health Sciences and Medicine, University of Lucerne, Lucerne, Switzerland; 5 Department of Orthopedics and Trauma, Luzerner Kantonsspital, Lucerne, Switzerland; 6 Service of Orthopaedics and Traumatology, Department of Surgery, EOC, Lugano, Switzerland; 7 Department of Trauma Surgery, Hand, Plastic and Reconstructive Surgery, University of Ulm, Ulm, Germany; 8 Department of General, Hand, and Trauma Surgery, City Hospital Triemli, Zurich, Switzerland; 9 Department of Orthopaedic Surgery and Traumatology, Kantonsspital Baselland (Bruderholz, Liestal, Laufen), Bruderholz, Switzerland; 10 Department of Trauma, Hand and Reconstructive Surgery, Friedrich-Schiller-University Jena, Jena, Germany

**Keywords:** Femoral neck fractures, Fractures, Implants, Femoral Neck System

## Abstract

**Introduction:**

Osteosynthesis of femoral neck fractures (FNFs) is an important treatment option, especially for younger patients. We aimed to assess the rate of early implant-related complications in FNF osteosynthesis using the Femoral Neck System (FNS).

**Patients and methods:**

Consecutive patients diagnosed with displaced or nondisplaced FNFs were treated with FNS in this prospective, observational, multicenter investigation. Patients were followed up for minimally 3 months and up to 12 months if radiologic bone union and no pain was not achieved beforehand. Predefined treatment-related adverse events (AEs, defined as implant failure, loss of reduction, iatrogenic fractures, deep infection, and surgical revision), radiologic bone union, and patient-reported Harris hip score (HHS) and EQ-5D-5L index score were assessed.

**Results:**

One hundred and twenty-five patients were included in the study. Thirty-eight (30.4%) fractures were displaced (Garden III and IV), and 37 (29.6%) were vertical fractures (Pauwels type III). Predefined treatment-related AE rate at 3 months was 8 patients, 6.4% (95% CI, 2.8–12.2), and at 12 months, 11 patients, 8.8% (95% CI, 4.5–15.2). Cumulative incidences of bone union were 68% at 3 months, 90% at 6 months, and 98% at 12 months. The mean changes of HHS and EQ-5D-5L index score between preinjury and at 12 months were -7.5 (95% CI, [ – 21.1] to [6.2]) and  – 0.03 (95% CI, [ – 0.21] to [0.15]), respectively; neither were statistically significant.

**Conclusion:**

The current study on osteosynthesis of FNFs with the FNS resulted in treatment-related complication rates of 6.4% (95% CI, 2.8–12.2) at 3 months and 8.8% (95% CI, 4.5–15.2) at 12 months. On average, patients returned to preinjury function and quality of life. The current study may also indicate that the conventional wisdom of treating stable FNF in patients aged between 60 and 80 years with osteosynthesis may need to be reconsidered.

**Registration:**

The study is registered with ClinicalTrials.gov (registration number: NCT02422355).

## Introduction

Femoral neck fracture (FNF) is a devastating disease and can have high complication rates following surgery. Although arthroplasty has been shown to be superior to osteosynthesis in terms of reoperation rate, function, and quality of life in older patients with displaced FNFs, osteosynthesis is still an important option in managing non-displaced FNFs for all age groups and in displaced FNFs in younger patients, where femoral head preservation is a priority [1].

Sliding hip screws (SHS) and cancellous screws (CS) are 2 commonly used implants for osteosynthesis in FNFs. Recent meta-analyses confirmed that the 2 methods resulted in similar reoperation rates, postoperative hip function, complication rates, and quality of life [2, 3].

Whether FNFs are managed with SHS or CS, the complication rate can be high, with reported reoperation rates of 18–27% [4–7] and a failure rate of up to 43% [8]. The Femoral Neck System (FNS) is a new generation of implant that reduces rotational displacement and provides angular stability; it is biomechanically superior to a fixation with 3 CS in unstable FNFs [9]. In 2022, multiple clinical studies and meta-analyses have been published on the FNS, with complication rates estimated between 9.2% and 13.3% [10–12].

The primary goal of this current study was to assess early implant-related complications (up to 3 months postoperatively); the secondary objectives were to assess bone union, as determined by the treating surgeons, hip function, and quality of life.

## Patients and methods

### Study design

This study was a prospective, observational, multicenter case series. Ethics approvals were obtained from the ethics commissions responsible for the study sites in 2017. Consecutive patient enrollment was conducted between October 2017 and December 2019; informed consent was received from all patients included in the study. This study is registered with ClinicalTrial.gov (for registration number, see the title page).

The FNS (Synthes GmbH, Oberdorf, Switzerland) received the CE mark in 2017. It is an angular stable device with a screw in screw technology to provide rotational stability. The small footprint of the device allows a minimally invasive insertion technique, which should result in better retention of viable bones. The dynamic design of the bolt allows it to slide along the plate barrel ("telescoping"), thus achieving a dynamic fixation of the femoral head [9]. The primary objective of the investigation was to assess the rate of predefined treatment-related complications within 3 months. The secondary objectives were to assess bone union, as determined by the treating surgeon, and to document patients' hip function and quality of life.

### Patient population, inclusion exclusion criteria

Patients 18 years old or older diagnosed with a FNF (AO/OTA type 31-B1:B3) to be treated with the FNS were included. The choice of treatment was based on the judgement of the individual treating surgeons. Patients with ipsilateral pertrochanteric (AO/OTA type 31-A1 and 31-A2), intertrochanteric (AO/OTA type 31-A3), or subtrochanteric fractures were excluded.

### Procedure and follow-up

All treatments were conducted according to the local standard of care; surgeries were performed according to the manufacturer's "Instructions for Use." Patients were followed up after surgery at 6 weeks (only if it was the local practice) and 3 months. Afterward, if the patient had achieved bone union and no pain at the operated site, the treating surgeon could decide whether further follow-up was required. Follow-up visits at 6- and 12-month were performed if radiologic union was not achieved or if there was persistent or increasing pain at the operated site.

### Data collection and outcome measures

Baseline data included sex, age, Charlson Comorbidity Index [13] , mechanism of injury, and fracture types according to the Garden [14] and Pauwels classification [15]. Surgical details recorded included time from injury to surgery, duration of surgery from skin incision to closure, and type of reduction.

The primary outcome measure was the treating surgeon-assessed rate of predefined treatment-related adverse events (AEs) within 3 months of surgery. These included (1) cut-out (penetration of the blade or screw through the femoral head into the joint with varus collapse), (2) cut-through (central penetration of the blade or screw into the joint and/or the lesser pelvis without varus collapse), (3) implant failure (breakage/bending), (4) iatrogenic fractures, (5) secondary displacement of the femoral head in relation to the implant, (6) clinical complications due to telescoping of the implant (movement of the barrel within its hole in the FNS plate in relation to the plate, resulting in a shortening of the femoral neck due to compression of the fracture), (7) deep wound infections, and (8) other AEs leading to surgical revision.

Secondary outcome measures included treating surgeons’ radiologic evaluation of bone union, quality of reduction assessed by an independent radiologist based on Garden's alignment index and Lowell's criteria [16, 17], and patient-reported Harris hip score (HHS) and EQ-5D-5L index score [18, 19]. The preinjury HHS and EQ-5D-5L were assessed retrospectively.

The HHS ranges from 0 to 100; higher scores indicate better function (poor, < 70; fair, 70 to < 80; good, 80 to < 90; and excellent, 90–100). The EQ-5D-5L index score ranges from  – 0.281 to 1 (higher score indicates better health) [19].

Safety analyses were performed using all data up to 12 months. Two senior surgeons reviewed the radiographs of patients who suffered predefined AEs to assess the potential cause of the AEs.

### Statistics

A sample size of 112 patients was determined based on the assumption of an AE rate of 15% and a dropout rate of 10% to allow an estimate precision of 14% (95% confidence interval [CI], 8–22%). Patient data were censored after bone union and no pain at operated site were achieved, and data collected afterward were not part of the analyses, except in the safety analysis, if a patient suffered an AE.

Data were reported as mean (standard deviation, SD) or median (interquartile range, Q1–Q3). For the primary outcome, the event rate of predefined treatment-related AEs occurring within 3 months and its 95% Clopper–Pearson CI were calculated. The bone union incidence analyses were performed using the cumulative incidence function with the Kaplan–Meier method [20]. The change in HHS and EQ-5D-5L index scores was analyzed using the mixed models for repeated measures (MMRM), where missing outcomes were compensated by modeling the within-patient correlation to provide information on missing outcomes. For HHS, up to 2 missing values were accepted and imputed by the mean value; an overall score was not calculated if more than 2 items were unanswered. The EQ-5D-5L index scores were weighted according to the German value sets; a missing value resulted in a missing total score.

Scatter plots of HHS were generated to visualize the potential relationship between age and functional outcome; a sensitivity analysis was performed using the final HHS scores (the scores at the time when bone union and no pain were achieved or at 12 months) of patients with complete follow-up. Box plots were generated to visualize the potential relationship between HHS and fracture types according to age groups.

Statistical testing using MMRM was conducted at the 2-sided 5% significance level. The event rate of all AEs occurring within 1 year (365 days) of the surgery was calculated with the 95% Clopper–Pearson CI.

## Results

**Fig. 1:**
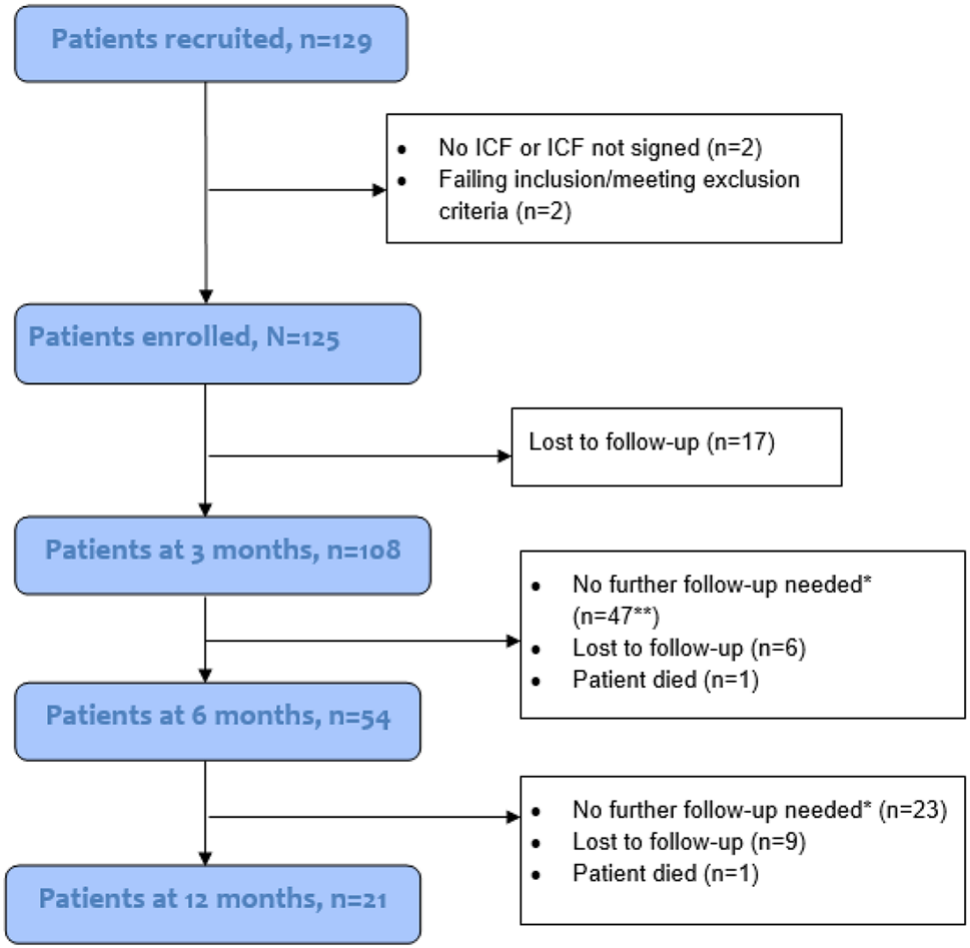
Patient enrollment and follow-up diagram. *Patients with bone union and no pain at a visit required no more follow-ups. **Including 1 patient who died of cancer between 3 and 6 months after surgery. Because this patient was considered as needing no further follow-ups at the 3-month visit, the patient was not counted as a lost-to-follow-up. One patient died of carcinoma between 3 and 6 months after surgery; 1 patient died of metastatic breast cancer between 6–12 months after surgery. Neither completed the planned visits

Eight clinics in 3 countries (Austria, Germany, and Switzerland) participated in the study and recruited 129 patients (Fig. 1). Four patients were not enrolled due to protocol deviations involving the informed consent process (*n* = 2) and ineligibility involving the inclusion/exclusion criteria (*n* = 2), resulting in a final enrolled patient population of 125.

The dropout rate at 3 months was 17 patients (13.6%); 3 patients died of cancer (carcinoma and metastatic breast cancer) after the 3-month follow-ups (Fig. 1). The number of patients to be followed up at each visit is shown in Fig. 1.

Baseline information is presented in Table 1: The mean (SD) age was 65.8 (18.0) years. Thirty-eight (30.4%) fractures were displaced (Garden III and IV), and 37 (29.6%) were vertical fractures (Pauwels type III).

**Table 1:** Baseline information and surgical details

Variable	Description	(*N* = 125)
Gender	Female, *n* (%)	71 (56.8)
	Male, *n* (%)	54 (43.2)
Age (years)	Mean (SD)	65.8 (18.0)
	18 to 59, *n* (%)	49 (39)
	60 to 79, *n* (%)	45 (36)
	80 to 99, *n* (%)	31 (25)
Charlson comorbidity index	Median (Q1–Q3)	1.0 (0.0–3.0)
Mechanism of injury	High energy trauma (e.g., motor vehicle), *n* (%)	23 (18.4)
	Low energy trauma (e.g., falls at home), *n* (%)	102 (81.6)
Garden fracture classification	Type 1: Stable fracture with impaction in valgus, *n* (%)	52 (41.6)
	Type 2: Complete but non-displaced, *n* (%)	35 (28.0)
	Type 3: Partially displaced with varus displacement but with contact between 2 fragments, *n* (%)	35 (28.0)
	Type 4: Completely displaced with no contact between the fracture fragments, *n* (%)	3 (2.4)
Pauwels classification	Type I: < 30 degree from horizontal, *n* (%)	25 (20.0)
	Type II: 30 to 50 degree from horizontal, *n* (%)	63 (50.4)
	Type III: > 50 degree from horizontal, *n* (%)	37 (29.6)
Time from injury to surgery (hours)	Median (Q1–Q3)	18.0 (8.0–32.0)
Duration of surgery, from incision to closing (minutes)	Mean (SD)	48.0 (15.27)
Type of reduction	Open, *n* (%)	4 (3.2)
	Closed, *n* (%)	120 (96.0)
	Missing, *n* (%)	1 (0.8)

*SD* standard deviation, *Q1–Q3* interquartile range

### Surgical details

The median (Q1–Q3) time from injury to surgery was 18 (8.0–32.0) hours. Closed reduction was performed in 120 out of 125 patients, and the mean (SD) operation time from skin incision to closure was 48.0 (15.27) minutes (Table 1). Plates with 1 hole were used in 118 out of 125 patients.

### Primary endpoint

Eight patients each suffered 1 predefined treatment-related AE within 3 months postoperatively, leading to a treatment-related AE rate of 6.4% (95% CI, 2.8–12.2) (Table 2).

**Table 2:** Occurrence of predefined treatment-related adverse events within 3 months

Adverse events	Total (*N* = 125)	
	*n*	% (95% CI)
Predefined treatment-related adverse event, all categories	8	6.4 (2.8–12.2)
Cut-out	0	0.0 (0.0–2.9)
Cut-through	2	1.6 (0.2–5.7)
Implant failure (breakage/bending)	2	1.6 (0.2–5.7)
Iatrogenic fractures	0	0.0 (0.0–2.9)
Secondary displacement of the femoral head in relation to the implant	2	1.6 (0.2–5.7)
Telescoping of the implant	1	0.8 (0.0–4.4)
Deep wound infection	0	0.0 (0.0–2.9)
Nonunion leading to surgical revision	1	0.8 (0.0–4.4)

### Secondary endpoints

#### Quality of reduction

The quality of reduction and its maintenance are summarized in Table 3. The postoperative quality of fracture reduction was mostly "anatomic" or "acceptable" according to the Garden's alignment index (*n* = 114, 91.2%) and the Lowell's criteria (*n* = 107, 85.6%). The reduction was well-maintained at 3 months postoperatively (Table 3).

**Table 3:** Quality of reduction and retention of reduction, assessment by an independent expert

Variable	Characteristic	Post-op (*N* = 125)	6 weeks (*N* = 114)	3 months (*N* = 103)	6 months (*N* = 43)	12 months (*N* = 19)
Garden's alignment index	Anatomic/acceptable	114 (91.2%)	106 (93.0%)	95 (92.2%)	38 (88.4%)	16 (84.2%)
	Borderline/unacceptable	11 (8.8%)	8 (7.0%)	8 (7.8%)	5 (11.6%)	3 (15.8%)
Lowell's criteria	Anatomic/acceptable	107 (85.6%)	92 (80.7%)	84 (81.6%)	31 (72.1%)	13 (68.4%)
	Borderline/unacceptable	14 (11.2%)	22 (19.3%)	16 (15.5%)	9 (20.9%)	4 (21.1%)
	Missing	4 (3.2%)	0 (0.0%)	3 (2.9%)	3 (7.0%)	2 (10.5%)

#### Bone union

**Fig. 2:**
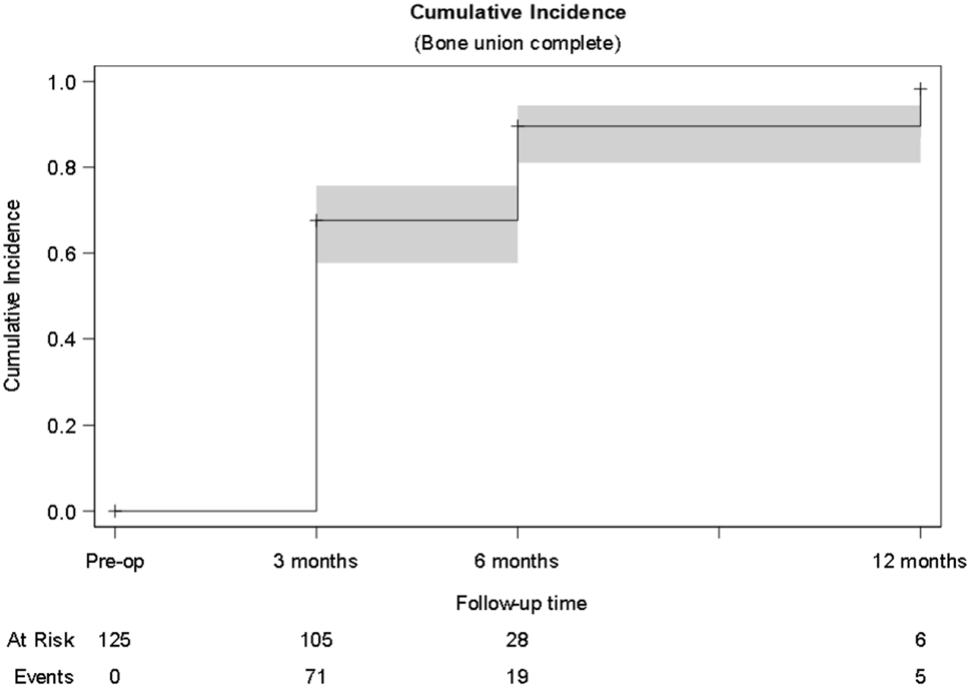
Cumulative incidence of bone union, assessment by the treating surgeon. Analysis of cumulative bone union incidence rates according to the at-risk populations. The number of patients at risk at a specific timepoint was defined as the number of patients who had not yet achieved bone union and were available for follow-up at that timepoint or later. The cumulative rates were 0.68 (95% confidence interval [CI], 0.58–0.76) at 3 months, 0.90 (95% CI, 0.81–0.94) at 6 months, and 0.98 (95% CI, 0.87–1.00) at 12 months

The cumulative incidence of bone union was 0.68 (95% CI, 0.58–0.76) at 3 months, 0.90 (95% CI, 0.81–0.94) at 6 months, and 0.98 (95% CI, 0.87–1.00) at 12 months (Fig. 2). At 12 months, bone union was achieved in 95 patients according to the treating surgeons. Only 1 patient was recorded to have persistent nonunion and was revised to total hip arthroplasty (Table 2).

#### Function and quality of life

Before injury, most of the patients had "excellent" hip function [median (Q1–Q3) HHS score, 100 (90.2–100.0)] (Table 4). At 6-week visits, patients overall scored significantly worse with a mean change from preinjury of  – 23.1 (95% CI, [ – 26.9] to [  – 19.3]). At 3- and 6-month visits, although the scores had improved, they were still statistically worse than at preinjury. At 12 months, with a mean change of  – 7.5 (95% CI, [ – 21.1] to [6.2]), the overall score was not significantly different from that at preinjury.

**Table 4:** Harris hip score and quality of life

	Score	Pre-injury (*N* = 125)	6 weeks (*N* = 114)	3 months (*N* = 103)	6 months (*N* = 43)	12 months (*N* = 19)
Harris hip score	Median (Q1–Q3)	100 (90.2–100.0)	70 (55.4–81.5)	82 (66.0–93.5)	82 (70.7–95.5)	94.4 (70.9–97.0)
	Change* (95% CI)		– 23.1 ([– 26.9] to [-19.3])	– 14.9 ([– 18.9] to [– 10.9])	– 10.8 ([– 15.1] to [– 6.5])	– 7.5 ([– 21.1] to [6.2])
(Missing)		6 (4.8%)	7 (6.1%)	7 (6.8%)	4 (9.3%)	1 (5.3%)
EQ-5D-5L index score	Median (Q1–Q3)	1.0 (0.83–1.00)	0.8 (0.70–0.91)	0.8 (0.74–1.00)	0.8 (0.76–0.92)	0.9 (0.79–1.00)
	Change* (95% CI)		– 0.16 ([– 0.22] to [-0.10])	– 0.09 ([– 0.14] to [– 0.05])	– 0.07 ([– 0.12] to [-0.01])	– 0.03 ([– 0.21] to [0.15])
(Missing)		5 (4.0%)	7 (6.1%)	6 (5.8%)	4 (9.3%)	1 (5.3%)

The Harris hip score ranges from 0 to 100, the higher the score, the better the function. Scores are considered poor (< 70), fair (70 to < 80), good (80 to < 90), and excellent (90–100)

^*^Change: Change from preinjury analyzed according to the mixed models for repeated measures. *CI *confidence interval

The EQ-5D-5L index scores showed a similar pattern of recovery: At 6-week, 3-month, and 6-month visits, the EQ-5D-5L index scores were significantly worse than at preinjury (Table 4). At 12 months, the mean change was  – 0.03 (95% CI, [ – 0.21] to [0.15]), not reaching statistically significant difference from the mean preinjury score.

#### Influence of age

**Fig. 3:**
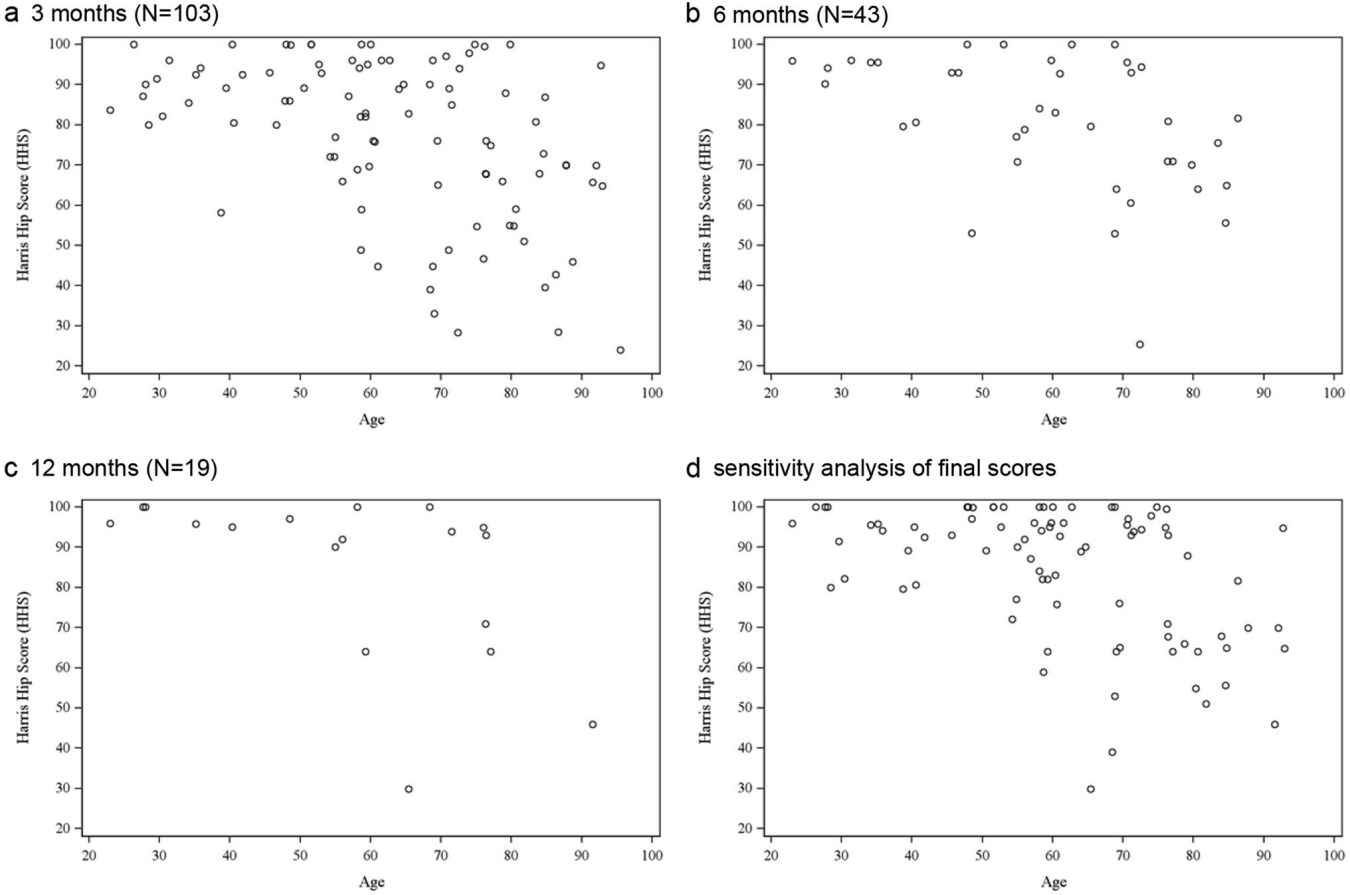
Functional outcome and age. Scatter plots of Harris hip scores (HHS) against age. **a** 3 months, **b** 6 months, **c** 12 months, **d** sensitivity analysis: final HHS scores of patients with complete follow-up

Figure 3a shows that, except for 1 extreme case, patients under roughly 58 years of age scored "good" or "excellent" in HHS categories at 3 months; patients 58-year-old or older, however, had scores scattered through the whole spectrum from around 25 to 100. Although the patterns are harder to discern at 6 and 12 months (Fig. 3b and c) due to the small sample sizes, they are consistent with the pattern seen in Fig. 3a. A sensitivity analysis of the final HHS in patients who completed the final follow-up showed similar results (Fig. 3d).

#### Influence of fracture type

**Fig. 4:**
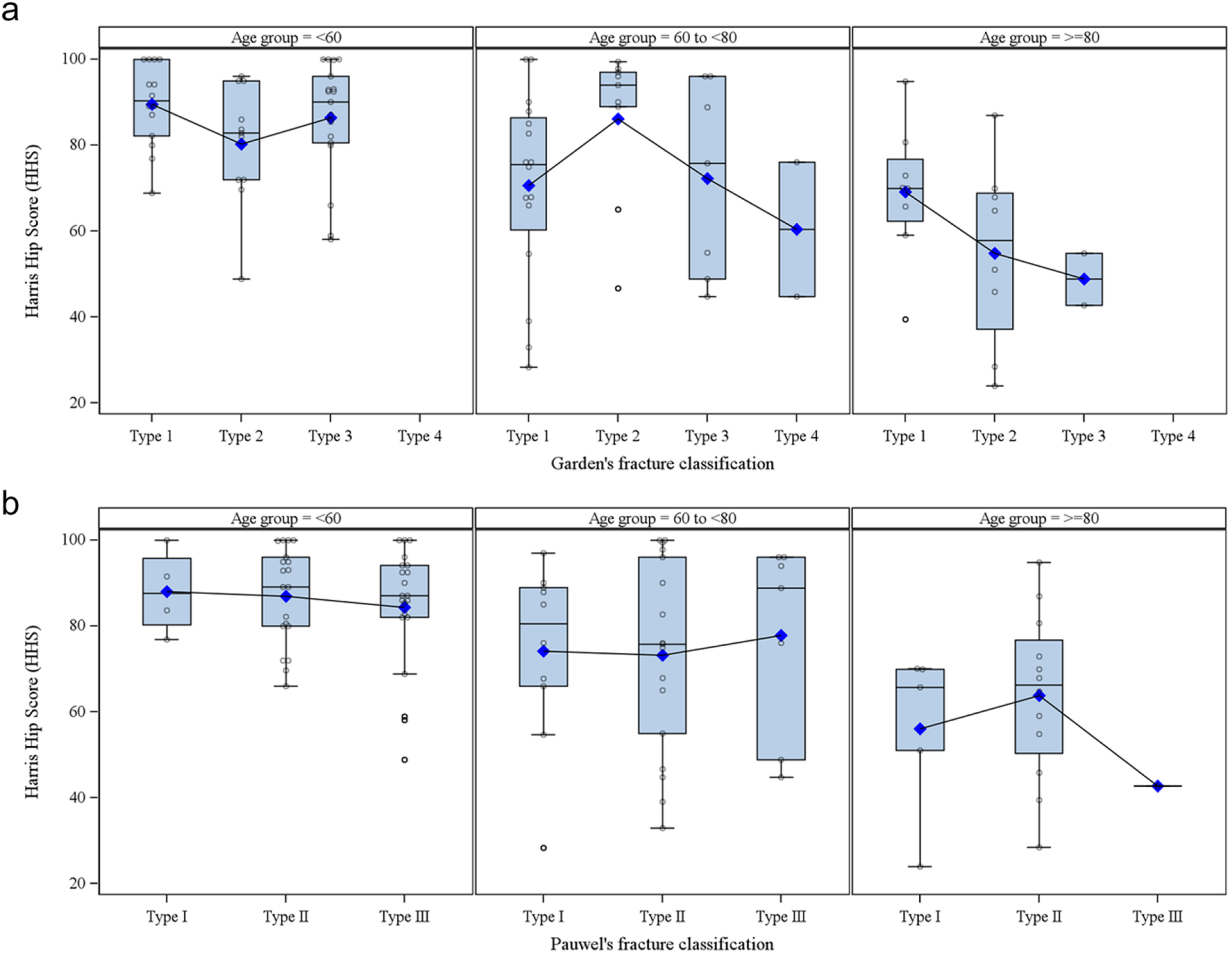
Functional outcome and fracture type. **a** Harris hip scores at 3 months after surgery according to Garden's fracture classification (*N* = 103). The lower/upper ends of the box represent quartile 1 and 3 of the Harris hip scores (HHS) of the age group. The lower/upper whisker represents the minimum/maximum (outliers disregarded) of the scores. The horizontal line within each box represents the median value, and the blue diamond represents the mean value. The circles represent individual HHS scores. **b** Harris hip scores at 3 months after surgery according to Pauwels fracture classification (*N* = 103). The lower/upper ends of the boxes represent quartile 1 and 3 of the Harris hip scores (HHS) of the age group. The lower/upper whiskers represent the minimum/maximum (outliers disregarded) of the scores. The horizontal line within each box represents the median value, and the blue diamond represents the mean value. The circles represent individual HHS scores

HHS results of the different age groups at 3 months were further analyzed by either Garden's (Fig. 4a) or Pauwels' (Fig. 4b) fracture types. As shown in Fig. 4a and b, a consistent pattern of how fracture types may influence the outcome is not clearly discernible using either classification.

#### Safety analysis

Eleven patients suffered 11 predefined AEs within 12 months after surgery, leading to a predefined AE rate of 8.8% (95% CI, 4.5–15.2). Among these patients, 9 reoperations were performed to remove the implant and/or revise to arthroplasty. A revision was recommended to 2 additional patients, but the patients either refused it or dropped out with unknown outcomes.

During a review of the radiographic images by two senior surgeons, it was determined:

- technical errors (over drilling, improper placement of the implant, unacceptable reduction) occurred in 3 cases,
- wrong indication (false diagnosis of Garden type III as Garden type I fracture in 2 elderly patients) was applied in 2 cases,
- a combination of factors (false presumption of non-eligibility of arthroplasty, technical error, and wrong indication) contributed to the AEs in 3 cases, and
- no obvious cause could be determined in 3 cases

Ten additional not predefined AEs were recorded within 12 months in 8 patients: 1 case each of bloody stool, purulent cholangitis, pulmonary embolism, pneumonia, pancreatitis, peripheral arterial disease, peri-implant fracture due to a fall, and 3 deaths mentioned earlier. In total, 18 patients suffered at least 1 AE within 12 months of surgery, leading to an all-AE event rate of 14.4% (95% CI, 8.8–21.8).

## Discussion

### Primary endpoint: treatment-related adverse events at 3 months

To capture a general impression of the clinical performance (up to 12 months) of the newly available FNS for osteosyntheses in FNFs, we enrolled 125 patients aged 23–100 years. The rate of predefined treatment-related AEs at 3 months was 6.4% (95% CI, 2.8–12.2), which is considerably lower than the assumption of 15% for sample size calculation. We also report a 1-year predefined AE rate of 8.8% (95% CI, 4.5–15.2). Due to the high dropout rate (13.6% patients at 3 months and 25.6% at 12 months), we suggested that the AE rates were likely in the upper range of the confidence intervals. Although many studies and meta-analyses on the performance of the FNS have been published recently, the heterogeneity of the study designs and the outcomes reported makes it difficult to compare the results. The closest comparison would be the studies published by Davidson et al. [11] and Schuetze et al. [10]. Davidson et al. reported an overall revision rate of 9.2% (average follow-up of 7 months); Schuetze et al. reported a surgical complication rate of 13.3% (mean follow-up of 13 months), with surgical complication defined as implant failure, hematoma, and implant-related infection.

Our mean (SD) surgical time (48.0 [15.27] minutes) was similar to a recently reported literature value of surgical time for the FNS (48.7 [15.4] minutes) [11]—both were shorter than the operative time reported in a meta-analysis for SHS plus (48.3–111 min) and on the low end compared to SHS or CS (43–61.6 min) [21].

### Age and treating femoral neck fracture with the Femoral Neck System

A gray area remains for the treatment of FNF patients between ages 60 and 80 [1]. Studying a group of 1,111 patients 55–70 years old, Bartels et al. reported a reoperation rate of 27% within 12 months after osteosynthesis versus 2.8% after arthroplasty [5]. In agreement with this perceived gray area, we observed that this "gray area" may also be reflected in functional outcomes, such as the HHS. In our study, patients around 58 years and younger scored mostly "good" or "excellent" in HHS categories, while patients aged between 58 and 80 years, similar to patients older than 80 years, had HHS scores scattered from around 25 to 100.

This result suggests that age, rather than fracture type, may have greater influence on the outcome of FNF osteosynthesis using the FNS. However, because our sample size calculation was not powered to address this question, further investigation will be necessary. Future prospective studies would be necessary to confirm this observation and should focus on understanding how other patient-related factors, such as comorbidities and substance use (e.g., smoking and alcohol), may influence the outcome in patients 60–80 years old.

### Bone union

The currently study reports a cumulative incidence of bone union of 68% at 3 months, 90% at 6 months, and 98% at 12 months in a population with 30.4% unstable and 29.6% vertical fractures. Although an exact bone union rate is difficult to calculate due to the study design, the currently reported bone union rates at 3 months and 12 months were faster and higher than the 67% fracture healing at 24 months reported for the FAITH trial, in which SHS and CS were used for osteosynthesis [6]. Similarly, in our series, only one nonunion occurred and required a revision to total hip arthroplasty—a much lower rate when compared to the 6% overall nonunion rate reported in the FAITH trial [6]. Of course, one must bear in mind that the two studies had different populations—the patients in FAITH trial were slightly older (mean age [SD], 72.1 [12.2] years). A future prospective comparative study will be necessary to compare the three implants directly.

### Return to function

In the current study, the difference between the preinjury and the 12-month HHS was not statistically significant; a comparison of EQ-5D-5L index scores at preinjury and 12 months yielded similar results. Therefore, the FNS-treated patients in the current study had likely returned to their preinjury status.

Multiple systematic review or meta-analyses have been published in 2022 on the FNS; all of them included only retrospective studies, suggesting that our study is likely the only prospective study in the FNS field [3, 11, 12, 22]. Due to the retrospective study design, very few previous studies have included patient-reported outcomes and none had collected preinjury patient outcomes. As a result, we could only compare our observation to the results reported in the FAITH-2 trial, even though the FAITH-2 population was different from ours, i.e., the FAITH-2 patient population was younger (mean age [SD] of 41.1 [12.4] years) but had more severe fractures (71% displaced fractures, i.e., Garden type III and IV fractures) [7]. In the FAITH-2 trial, in contrast to our results, patients did not return to the preinjury function and health-related quality of life scores at 12 months [7]. The differences may be due to the different patient populations, fracture characteristics, implants used (i.e., the FNS versus SHS and CS), and/or the study designs. We have considered whether our study design, with only 43 patients followed up at 6 months and 19 at 12 months, could have explained the difference in outcome, but since these patients followed up to 6 and 12 months were patients that did not achieve earlier healing (bone union and recovery from pain), they were more likely to have worse scores than the average patients in the current study. We therefore consider this was unlikely the cause of the difference.

### Strengths and limitations

The limitation of the current study lies in its observational study design where patients were followed up as they would normally be treated. As a result, the bone union rates are difficult to compare to other reports. In addition, the study suffered a high dropout rate of 13.6% at 3 months, which means that, the actual AE rates, i.e., 6.4% (95% CI, 2.8–12.2) at 3 months and 8.8% (95% CI, 4.5–15.2 at 12 months, were more likely in the upper range of the confidence interval.. The study may also have suffered from "expertise bias" because the centers were considered clinics particularly experience in this type of surgeries. The strength of the current study lies in its prospective design and the inclusion of predefined complications. As a multicenter study without age and fracture type restrictions, the results can be applied broadly to different patient populations.

## Conclusion

In summary, in our prospectively enrolled patient population treated with the FNS followed up to 12 months, the predefined, treatment-related AE rate was 6.4% (95% CI, 2.8–12.2) at 3 months and 8.8% (95% CI, 4.5–15.2) at 12 months. On average, patients were able to return to preinjury function and quality of life. Disregard the fracture characteristics, patients aged 60–80 years had worse HHS at 12 months than younger patients; therefore, the conventional wisdom of using osteosynthesis to treat older patients with stable FNF may need to be reconsidered.

## Data Availability

Individual patient data will not be available. Individual researchers may contact the corresponding author for access to the original, aggregated and anonymized datasets for research purposes.
